# Adsorption Behavior and Dynamic Interactions of Anionic Acid Blue 25 on Agricultural Waste

**DOI:** 10.3390/molecules27051718

**Published:** 2022-03-06

**Authors:** Ensan Waatriah E. S. Shahrin, Nur Alimatul Hakimah Narudin, Nurulizzatul Ningsheh M. Shahri, Sera Budi Verinda, Muhammad Nur, Jonathan Hobley, Anwar Usman

**Affiliations:** 1 Department of Chemistry, Faculty of Science, Universiti Brunei Darussalam, Jalan Tungku Link, Bandar Seri Begawan BE1410, Brunei; 20m8522@ubd.edu.bn (E.W.E.S.S.); 20m2042@ubd.edu.bn (N.A.H.N.); 20h8540@ubd.edu.bn (N.N.M.S.); 2 Biomedical Graduate Program, Faculty of Medicine, Universitas Diponegoro, Tembalang Campus, Semarang 50275, Indonesia; serabudiverinda@gmail.com; 3 Center for Plasma Research, Integrated Laboratory, Universitas Diponegoro, Tembalang Campus, Semarang 50275, Indonesia; nur.cpr@gmail.com; 4 Department of Biomedical Engineering, National Cheng Kung University, No. 1 University Road, Tainan City 701, Taiwan; jonathan.hobley@gmail.com

**Keywords:** acid blue 25, adsorption equilibrium, kinetics, mass transfer, adsorption mechanism, pomelo pith

## Abstract

In this study, adsorption characteristics of a negatively charged dye, Acid Blue 25 (AB25), on pomelo pith (PP) was studied by varying the adsorption parameters, with the aim of evaluating the adsorption mechanism and establishing the role of hydrogen bonding interactions of AB25 on agricultural wastes. The kinetics, intraparticle diffusion, mechanism, and thermodynamics of the AB25 adsorption were systematically evaluated and analyzed by pseudo-first-order and pseudo-second-order kinetic models, the Weber–Morris intraparticle and Boyd mass transfer models, the Langmuir, Freundlich, Dubinin–Radushkevich, and Temkin isotherm models, and the Van’t Hoff equation. It was found that AB25 adsorption followed pseudo-second-order kinetics, governed by a two-step pore-volume intraparticle diffusion of external mass transfer of AB25 onto the PP surface. The adsorption process occurred spontaneously. The adsorption mechanism could be explained by the Langmuir isotherm model, and the maximum adsorption capacity was estimated to be 26.9 mg g^−1^, which is comparable to many reported adsorbents derived from agricultural wastes. Changes in the vibrational spectra of the adsorbent before and after dye adsorption suggested that AB25 molecules are bound to the PP surface via electrostatic and hydrogen bonding interactions. The results demonstrated that the use of pomelo pith, similar to other agricultural wastes, would provide a basis to design a simple energy-saving, sustainable, and cost-effective approach to remove negatively charged synthetic dyes from wastewater.

## 1. Introduction

Soil and water environmental issues caused by the discharge of industrial wastewaters into water systems have become a global concern. In particular, the detrimental effects of the pollutants, such as heavy metals and synthetic dyes, on the entire ecosystem and human health have prompted the drive to develop effective approaches to remove the pollutants from wastewaters [1,2]. A variety of physical, chemical, and biological approaches, such as membrane filtration [3], flocculation [4], advanced oxidation [5,6], ozonation [7], adsorption [8,9,10,11], ion exchange [12], Fenton processes [13], and biodegradation [14] have been explored to remove heavy metal and dye effluents from wastewater. Among these promising methods to remove the pollutants, adsorption has attracted much attention because it is simple, cheap and practical to implement and can be handled even by a small-scale industry using non-toxic materials. Another key advantage is the wide variety of possible adsorbents that can be selected, ranging from naturally available materials to well-designed synthetic materials. Among them, agricultural wastes offer several benefits in terms of their cost effectiveness, their wide variety of functional groups, and their ability to scavenge heavy metal and synthetic dyes.

The cost of the adsorbents and their maximum adsorption capacity ($Q_{m}$) are two of the important criteria used to evaluate their potential for cost-effective wastewater remediation. Industrial waste, agricultural waste, and biomass are among the most promising alternative materials used as adsorbents [10,11]. This is due to their low cost, reduced environmental footprint, and their availability. Moreover, agricultural wastes have plenty of functional groups which can readily chelate heavy metals or interact with organic dyes. In addition, they can be chemically modified to optimize the surface concentration of specific functional groups, and they can be carbonized followed by thermal or chemical activation to form activated carbon, which is the most effective adsorbent. By adapting and modifying the adsorption process, a variety of agricultural wastes have been explored as adsorbents [11,15,16,17,18].

One of the common toxic acidic dyes found in industrial wastewater, which could potentially contaminate ecosystems is Acid Blue 25 (AB25) [19]. In recent years, adsorptive removal of AB25 from aqueous solution has been investigated by exploring different types of adsorbents such as polymer-clay nanocomposites [20], pectin [21], activated carbons [22,23], and agricultural wastes. These include banana peel and durian peel [11], sawdust [15], *Shorea dasyphylla* sawdust [24], oak sawdust [15], cempedak durian peel [25], rambutan seed [26], hazelnut shells [15], shrimp shells [27], peach seed [28], plant leaves [29,30], and soybean waste [31]. Many of the adsorbents have been demonstrated to successfully remove AB25 from water systems with high efficiency. Despite this abundance of work, a detailed description of the adsorption characteristics of AB25, a negatively charged dye, remains a relatively uncharted area of research. Moreover, interactions between the dye and adsorbents, which are responsible for the adsorption of AB25 on agricultural wastes, have never been comprehensively discussed. Recently, it has been pointed out that low methoxy pectin isolated from pomelo pith (PP) shows the highest $Q_{m}$ of AB25 due to the formation of hydrogen bonds with the galacturonic units of pectin strands which form gels in aqueous solution [21]. To further expand on the important role of hydrogen bonds, in the current study, the adsorption mechanism and hydrogen bonding interactions of AB25 on PP as a representative model of agricultural waste-based adsorbents are investigated. The objective of this work is therefore focused on assessing the adsorptive removal of AB25 on PP and its adsorption characteristics, including kinetics, isotherm, mechanism, rate-limiting step, and thermodynamics, and comparing these results with those for adsorption on pectin isolated from the pomelo pith and on other agricultural wastes. The adsorption behavior of AB25 was also investigated with respect to functional groups, pore size distribution, and other surface characteristics of PP. Intermolecular interactions between AB25 and PP were simulated using ab initio calculations using Gaussian basis sets.

## 2. Results and Discussion

### 2.1. The Functional Groups and Surface Morphology of PP

**Figure 1:**
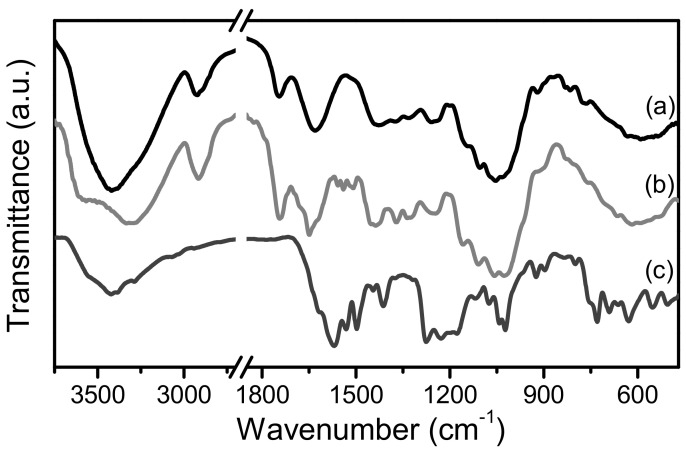
The FTIR spectra of (**a**) PP before AB25 adsorption, (**b**) PP after AB25 adsorption, and (**c**) AB25.

FTIR spectra of PP along with that of AB25 are shown in Figure 1. Before adsorption, PP showed vibrational bands at 3410, 2923, and 2852 cm^−1^ which are assigned to OH, NH, and CH vibrations. Those at 1748, 1349, and 1050 cm^−1^ are assigned to carbonyl C=O, C–C, and C–O stretching vibrations, whereas the bands at 1632 and 1413 cm^−1^ are assigned to the asymmetric and symmetric stretching vibrations of carboxylic acid (COOH) groups, respectively. These vibrational bands most likely originate from the functional groups of cellulose, hemicellulose, pectin, and lignin contained in PP [18,32,33]. Before adsorption, AB25 has bands at ~1590 and 1575 cm^−1^ (C=O stretch on anthrone), 1532 cm^−1^ (aromatic C=C), 1500 cm^−1^ (aromatic C=C), 1415 cm^−1^ (S=O^…^Na stretch), 1280 cm^−1^ (C–N stretch on aromatic amine), 1230 cm^−1^ (C–N stretch), 1180, 1040, and 1020 cm^−1^ (SO_3_Na) [11,34].

Upon AB25 adsorption, the FITR spectrum of PP in general showed similar features with those before AB25 adsorption with a few changes. In particular, there are changes in relative peak intensity of the vibrational modes of C–O, C=O, and COOH groups of PP. In the fingerprint region, both spectra show broad bands at 489–840, 930–1200, and 1211–1476 cm^−1^, and sharp intense bands at 1627, 1653, and 1744 cm^−1^. These spectral features could be broadly assigned to the superposition of the vibrational spectra of PP and adsorbed AB25, with expected spectral shifts due to intermolecular interactions between PP and AB25. It is interesting to note that the OH vibrational band of PP before adsorption (3410 cm^−1^) split into two bands at 3040 and 3568 cm^−1^ upon AB25 adsorption. This suggests that there are hydrogen bonding interactions between the OH groups of PP with functional groups of AB25 which play a key role in stabilizing adsorption of the dye on the adsorbent surface.

**Figure 2:**
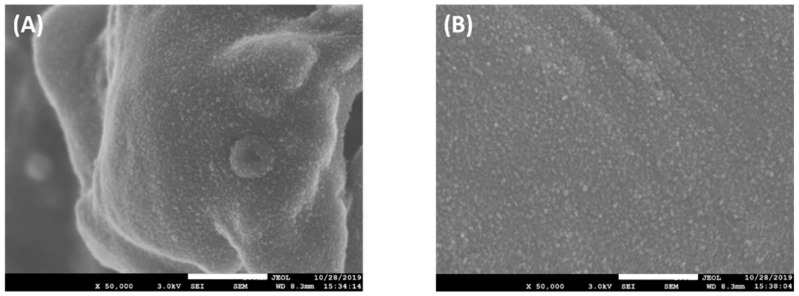
The SEM images of the PP surface (**A**) before and (**B**) after adsorption of AB25. All images were recorded at 50,000× magnification, and the scale bars represent 500 nm.

Figure 2 shows the SEM images of the PP before and after AB25 adsorption. These images suggest that the PP surface becomes smoother after adsorption. The BET surface area and total pore volume of PP were 12.3 m^2^ g^−1^ and 0.009 cm^3^ g^−1^, which is larger than those of other agricultural wastes such as banana peel and durian peel (7.5 m^2^ g^−1^ and 0.006 cm^3^ g^−1^, respectively) [11]. The surface area and total pore volume of PP after AB25 adsorption were reduced to 11.1 m^2^ g^−1^ and 0.006 cm^3^ g^−1^.

### 2.2. Adsorption Characteristics of AB25 on PP

**Figure 3:**
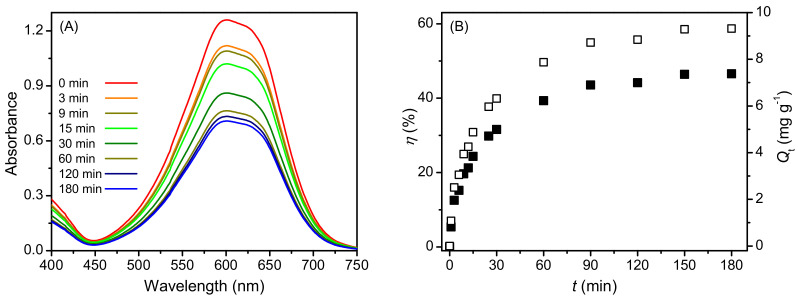
(**A**) Absorption spectra of AB25 on PP at different contact times, and (**B**) the adsorption efficiency (η; ■) and adsorption capacity (Q_t_; □) of AB25 as a function of contact time.

Adsorption of AB25 is manifest as a decrease in the absorbance of the remaining solution. Based on the absorption spectra of 20 cm^3^ solution of AB25 with an initial concentration, $C_{0}$ = 1.20 × 10^−4^ mol dm^−3^, in the presence of 25 mg PP, as shown in as shown in Figure 3A, the adsorptive efficiency (η) and the adsorption removal capacity ($Q_{t}$) as a function of contact time are calculated and presented in Figure 3B. It can be seen that the values of η and $Q_{t}$ of AB25 adsorption on PP increased nonlinearly with the contact time, and an equilibrium state was achieved with a contact time of 120 min with η and $Q_{t}$ being 43% and 9.32 mg g^−1^, respectively. It was also demonstrated that this equilibrium value increased nonlinearly with the adsorbent dosage (see Supplementary Information Figure S1). In this current study, PP showed a limited maximum removal efficiency of 85%. In comparison, pectin derived from PP could completely remove AB25 from the aqueous solution under the same experimental conditions [21].

**Figure 4:**
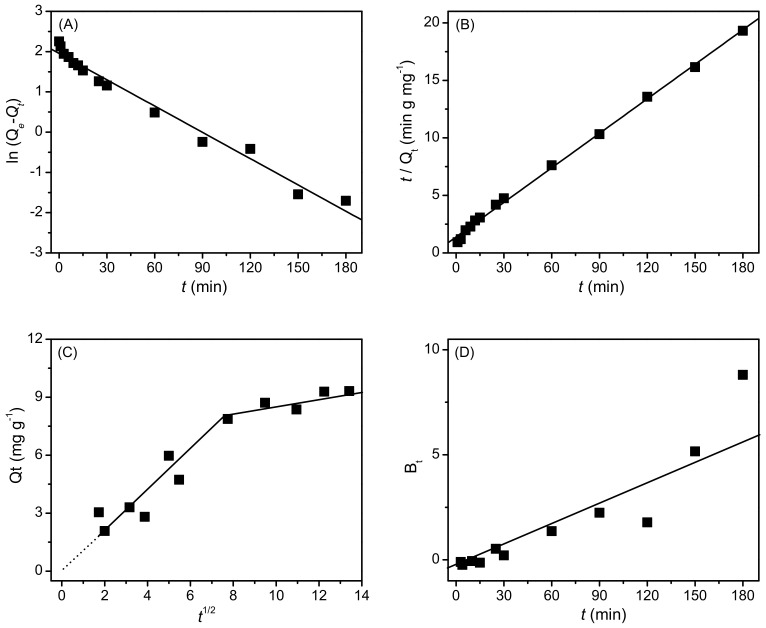
Adsorption kinetic data of AB25 on PP fitted with (**A**) Lagergren pseudo-first order, (**B**) pseudo-second order, (**C**) Weber–Morris intraparticle diffusion, and (**D**) Boyd diffusion models.

Simulations of Lagergren pseudo-first order and pseudo-second order on the adsorption kinetics of AB25 are shown in Figure 4A,B. Both kinetics models fitted well with the experimental data, but the regression coefficient ($R^{2}$) value of pseudo-second order (0.998) is higher than that of pseudo-first order (0.980), suggesting that the former model is better to explain the adsorption kinetics of AB25 on PP. Based on the best fit of the pseudo-second order model, the adsorption capacity at the equilibrium state ($Q_{e}$) was estimated to be 9.98 mg g^−1^, which is approximately reproduced the experimental results. The adsorption rate, $k_{2}$, of AB25 on PP was 0.007 g mg^−1^ min^−1^, suggesting low affinity of PP towards the acidic dye.

Simulation of the Weber–Morris intraparticle model on the adsorption kinetics data suggested a fast diffusion of dye onto the PP surface with a diffusion rate of 1.06 mg g^−1^ min^−1/2^ within the first 60 min of contact time. This was followed by a slower diffusion with a rate of 0.138 mg g^−1^ min^−1/2^ associated with achieving the equilibrium state. The zero y-intercept of the best fit indicated that the thickness of the boundary layer was negligible (close to zero) and that the AB25 adsorption on PP was governed by a diffusion controlled rate-limiting process (Figure 4C) [35,36]. In addition, the straight line of Boyd plot which nearly passes through the origin suggests that the external mass transfer kinetics is controlled by intraparticle diffusion.

**Figure 5:**
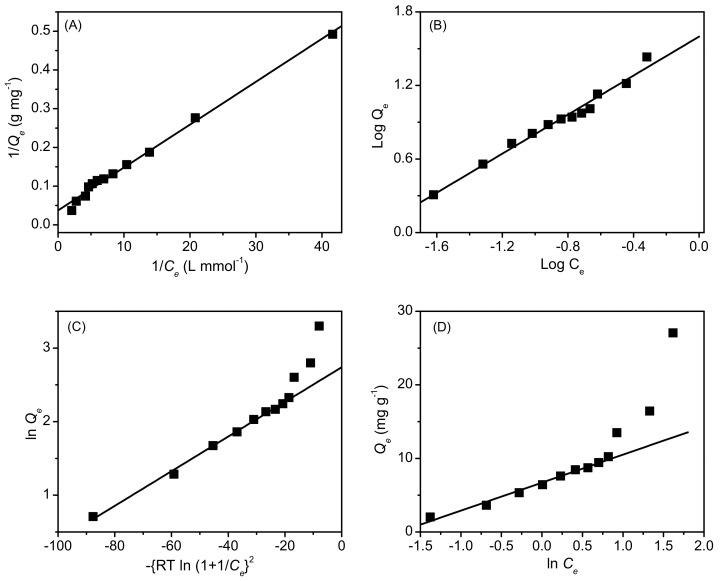
Adsorption isotherm data of AB25 on PP fitted with (**A**) Langmuir, (**B**) Freundlich, (**C**) Dubinin–Radushkevich, and (**D**) Temkin isotherm models.

As shown in Figure 5, the Langmuir and Freundlich isotherm models could simulate the experimental data well. In contrast, the Dubinin–Radushkevich and Temkin models showed high discrepancies between the simulated values and the experimental data, especially at high dye concentrations. The fitting parameters of the simulation best fits are presented in Table 1, indicating that the Langmuir model is the most suitable to explain the adsorption mechanism and distribution of AB25 as a homogeneous monolayer on the PP surface. As AB25 is negatively charged, one could consider that the dye is bound to the PP surface via electrostatic and hydrogen bonding interactions, and this is supported by shifts in the AB25 and PP bands in the FTIR spectra.

**Figure 6:**
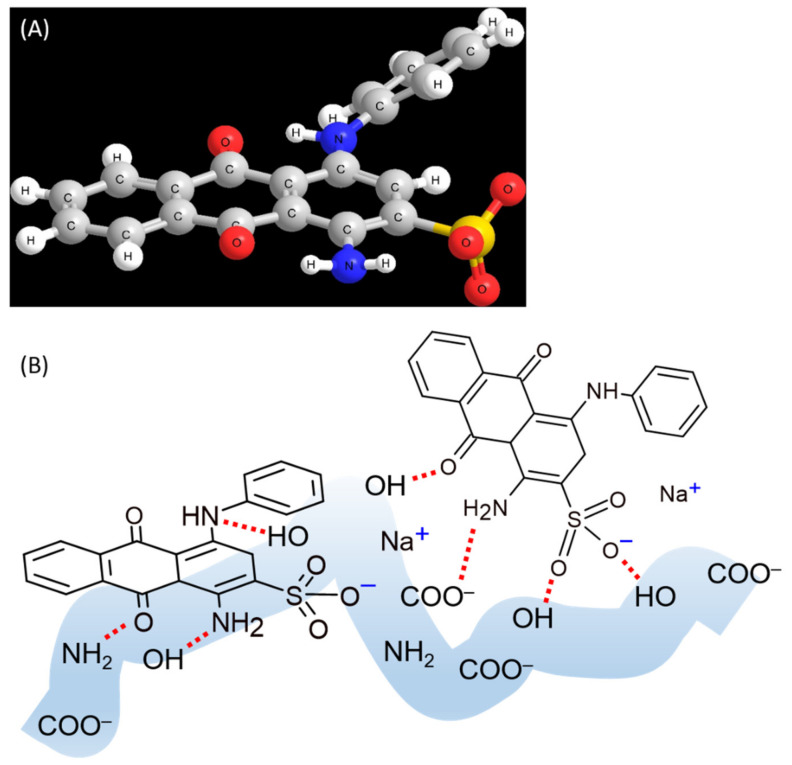
(**A**) The structure of AB25 optimized using Chem3D (the force field MMF94, 500 iterations, and the minimum RMS gradient 0.1), and (**B**) possible adsorption geometries of AB25 through hydrogen bonding interactions with the OH, NH_2_, C=O, and COO^−^ groups on the PP surface.

To exemplify this interpretation, intermolecular interactions between AB25 and PP in this study were simulated using ab initio calculations using Gaussian basis sets. The structure of AB25 was optimized in Chem3D. The energy minimization was performed using the MMF94 force field. The number of iterations was 500 and the minimum RMS gradient was 0.1. As shown in Figure 6A, it can be seen that AB25 has a relatively planar structure with a slightly out of plane with the secondary amine group twisted with respect to the phenyl ring. Two choices therefore exist for adsorption. The molecule can either lay flat allowing hydrogen-bond interactions through all amine, carbonyl, and sulfonate groups, albeit with a less than favorable geometry, or it could position itself with the more accessible 1° amide and the adjacent carbonyl and sulfonate pointing to the PP surface with a more favorable geometric conformation. The interactions which govern adsorption mechanisms of AB25 were mainly hydrogen bonding interactions involving the OH, NH_2_, C=O, and COO^−^ groups on the PP surface, as schematically illustrated in Figure 6B.

### 2.3. Comparison of the $Q_{m}$ of AB25

The $Q_{m}$ of AB25 on PP was estimated to be 26.9 mg g^−1^ based on the best fit of the Langmuir isotherm mode. The adsorption processes of AB25 reported were in batch mode, and in principle the $Q_{m}$ was calculated with respect to dried mass of the adsorbents. The $Q_{m}$ for the removal of AB25 on PP is listed and compared with many different adsorbents reported in the literature. As summarized in Table 2, the $Q_{m}$ for the removal of AB25 on PP was higher than kaolin [37], and it was comparable to those on other reported adsorbents, such as *Shorea dasyphylla* sawdust [24], pine sawdust [15], cempedak durian peel [25], oak sawdust [15], and rubber leaves powder [29]. However, it was lower compared to rambutan seed (*Nephelium lappaceum*) [26], walnut sawdust [15], soybean waste [31], *Azolla pinnata* [31], hazelnut shell [15], banana peel [11], *Ficus rasemosa* leaves powder [30], durian peel [11], peach seed powder [28], *Aspergillus oryzae* [38], egg shell modified activated carbon [23], *Ceratophylum demersum* [14], *Potamogeton pusillus* [14], activated carbon [22], pectin [21], and chitosan-activated carbon composite [39].

We note that the literature values of $Q_{m}$ were reported at different pHs, depending on the adsorbent. In most of adsorption studies many authors proved that the optimum removal was obtained at pH 2–4 which is below the pH of point of zero charge (pH_pzc_) of the adsorbents, making the adsorbents’ surface positively charged and favorable for adsorption of negatively charged synthetic dyes. It has been pointed out that the pH progressively affects the adsorption behavior of AB25 on adsorbents derived from agricultural wastes [15,39]. Moreover, based on our experience, changing the medium pH modifies, not only the ionic state and surface properties of PP adsorbent, but also the ionic state, intermolecular interactions, solubility, and aggregation of AB25. In this study, absorption of AB25 on PP was carried out using an un-buffered aqueous solution of AB25 with a known mass of PP, for which the “ambient” pH is nearly neutral (pH ~6.7), and which is above the pH of point of zero charge (pH_pzc_) of the adsorbent (4.23). At this pH, AB25 is in its anionic form (pKa ~1), whereas the aromatic carboxylic acid (–COOH; pKa 4.8) and primary and secondary amines (–NH_2_, –NH–; pKa < 4.6) of PP are naturally deprotonated [40,41]. This offers an interpretation that adsorption of AB25 relies solely on hydrogen bonding interactions with carboxylic acid, amines, hydroxyl (–OH) and other hydrogen bonding active functional groups on the PP surface. Considering that that adsorption of AB25 could be accelerated by electrostatic attraction to the deprotonated form of those functional groups, the difference in $Q_{m}$ values can be partly attributed to differences in pH. In addition, the surface area and pore size of any adsorbent could be modified in solutions with different pHs, which could lead to different adsorption capacities of the adsorbent. However, such a comparison at least provides a broad picture of adsorption characteristics of AB25 onto various adsorbents derived from agricultural wastes.

### 2.4. Thermodynamic Parameters of AB25 Adsorption

As shown in Table 1, the Langmuir isotherm constant, $K_{L}$, for the adsorption of AB25 on PP at 25 °C, is 3.36 dm^3^ mmol^−1^. Similar experiments were performed at different temperatures in the range 293–313 K. The estimated $K_{L}$ at each temperature was converted to dimensionless equilibrium constant, $K_{e}^{0}$, and the Gibbs free energy ($\Delta G_{\mathrm{ads}}$) was calculated based on Van’t Hoff equation, $\Delta G_{\mathrm{ads}}=-\mathrm{RT}\ln K_{e}^{0}$. It was found that $K_{e}^{0}$ increased and, hence, $\Delta G_{\mathrm{ads}}$ decreased, as temperature increased (see Table 3). This result suggests that adsorption of AB25 onto PP is spontaneous, and that the adsorption is energetically more favorable at higher temperatures. Based on the linear plot of $\Delta G_{\mathrm{ads}}$ against temperature (see Supplementary Information Figure S2), the enthalpy (ΔH) and entropy (ΔS) were then estimated to be 10.4 kJ mol^−1^ and 52 J mol^−1^ K^−1^, respectively, suggesting that the adsorption process is endothermic. It is interesting to note that ΔS of AB25 adsorption on PP is higher compared to that on pectin (47 J mol^−1^ K^−1^), but it is much smaller compared with those on other agricultural wastes, such as BP and DP (8.02 and 0.82 kJ mol^−1^ K^−1^, respectively). These results suggest that the PP surface is slightly less energetically stable than the pectin surface, but more stable than the surfaces of other agricultural wastes. In other words, upon AB25 adsorption, it was easier for the PP surface to become irregular as compared with pectin [42,43].

## 3. Materials and Methods

### 3.1. Chemicals

AB25 sodium salt (C_20_H_13_N_2_O_5_SNa) with a molecular weight of 416.38 g mol^−1^ and a molar decadic extinction coefficient of 1.05 × 10^4^ dm^−3^ mol^−1^ cm^−1^ at 600 nm, was purchased from Sigma–Aldrich Co. (St. Louis, MO, USA), and used as received. Aqueous solutions containing different concentrations of AB25 were prepared and the dye concentration was estimated based on the UV–visible absorption spectrum, measured on a Shimadzu UV-1900 (Shimadzu, Tokyo, Japan), in the range of 200–800 nm, using 1 cm path length cuvette.

PP was obtained from pomelo fruit by separating it from the fruit meat and flavedo. It was then chopped into small pieces and dried at 40 °C in an oven. After drying, the PP was ground into a course powder using a blender, sieved through 212 μm mesh, and used as an adsorbent.

### 3.2. Characterizations

The available functional groups on PP and intermolecular interactions of AB25 on the PP surface were determined based on its vibrational spectrum recorded on an FTIR spectrometer (IR Prestige-21, Shimadzu, Tokyo, Japan). Its surface morphology was characterized by scanning electron microscopy (SEM) using a JEOL JSM-7600F (JEOL, Tokyo, Japan). The surface characteristics, including Brunauer–Emmett–Teller (BET) surface area and Barrett–Joyner–Halenda (BJH) pore size distribution, were measured on a surface analyzer (ASAP 2460, Micromeritics, Norcross, GA, USA).

### 3.3. Adsorption of AB25 of PP

Mixture of AB25 stock solutions with a known mass of PP were gently shaken using an orbital shaker. The amount of adsorption was evaluated at different contact times. Typically, 20 cm^3^ of AB25 solution with an initial concentration, $C_{0}$ = 1.20 × 10^−4^ mol dm^−3^, was mixed with 25 mg of PP in conical flasks. The colloidal mixtures were shaken at room temperature for different contact times, t, from 0 to 240 min, followed by centrifugation. The solutions were separated, and their UV–visible absorption spectra were measured. In other experiments, the initial concentration of AB25 was varied from 0.48 × 10^−4^–9.60 × 10^−4^ mol dm^−3^. A total of 20 cm^3^ of these solutions was mixed with 25 mg PP and the adsorption was terminated after 180 min of contact time. The effect of adsorbent dosage was determined from the adsorption from 20 cm^3^ AB25 solution (1.20 × 10^−4^ mol dm^−3^) mixed with 25–200 mg PP. The thermodynamics of the adsorption process were investigated from a mixture of 20 mL AB25 solution (1.20 × 10^−4^ mol dm^−3^) with 25 mg PP, in the temperature range of 20–40 °C. All the adsorption experiments were carried out in duplicate, and all the collected data were combined and analyzed.

In every case, the residual concentration ($C_{t}$) was determined based on the absorption spectrum of the remaining solution. The adsorptive efficiency (η) was calculated from, $\eta \;\left(\%\right)=100\left(C_{0}-C_{t}\right)/C_{0}$, and the adsorption removal capacity ($Q_{t}$) was estimated using $Q_{t}=\left(C_{0}-C_{t}\right)V/M$; where M is the mass of PP, and V is the volume of AB25 solution, respectively.

The adsorption kinetics of AB25 on PP was carefully monitored at early contact times from 0 to 180 min. The time-dependent $Q_{t}$ data were analyzed using Lagergren pseudo-first order and pseudo-second order reaction laws with $k_{1}$ and $k_{2}$ being the respective rate constants [44];

(1) $$\ln\left(Q_{e}-Q_{t}\right)=\ln Q_{e}-k_{1}t$$

(2) $$t/Q_{t}=1/k_{2}Q_{e}^{2}+t/Q_{e}$$

To determine the rate-limiting step of in the adsorption of AB25, the same $Q_{t}$ data were also simulated using homogenous surface diffusion models, including the Boyd diffusion and the Weber–Morris pore-volume intraparticle models [45,46,47,48];

(3) $$B_{t}=-0.4977-\ln\left(1-Q_{t}/Q_{e}\right)$$

(4) $$Q_{t}=k_{i}t^{1/2}+C$$

where $B_{t}$ is Boyd constant of the AB25 adsorption, $k_{i}$ is the rate constant of intraparticle diffusion, and C is the thickness of the boundary layer.

The adsorption isotherm of AB25 on PP was analyzed using Langmuir, Freundlich, Dubinin–Radushkevich, and Temkin isotherm models [49];

(5) $$1/Q_{e}=1/Q_{m}K_{L}\times 1/C_{e}+1/Q_{m}$$

(6) $${\mathrm{Log}\;Q}_{e}=\frac{1}{n}{\mathrm{Log}\;C}_{e}+{\mathrm{Log}\;K}_{F}$$

(7) lnQe=lnQm−βRTln1+1Ce2

(8) Qe=RTbTln KT+ln Ce

where R is the gas constant, and T is absolute temperature. The physical quantities governing the adsorption process, including the maximum adsorption capacity ($Q_{m}$), the Langmuir, Freundlich, and Temkin isotherm constants ($K_{L}$, $K_{F}$, and $K_{T}$), Freundlich and Temkin exponent factor (n and $b_{T}$), the Dubinin–Radushkevich mean free energy change (β), were determined based on the best fits of the isotherm models on the adsorption isotherm data. The mean adsorption energy, E (kJ mol^−1^), was calculated using $E={\left(2\beta \right)}^{-1/2}$.

The thermodynamics of AB25 adsorption on PP was evaluated by calculating dimensionless equilibrium constant, $K_{e}^{0}$, [50];

(9) $$K_{e}^{0}=K_{L}\times \left[\mathrm{AB}25\right]/\gamma$$

where $\left[\mathrm{AB}25\right]$ is the concentration (1.20 × 10^−4^ mol dm^−3^), and thus γ, which is the activity coefficient of solution, was approximated to be unitary

## 4. Conclusions

In this study, the adsorptive removal and dynamic interactions of AB25, a negatively charged dye, on pomelo pith (PP) was investigated across different parameters. It was found that the adsorption kinetics was pseudo-second order with a rate constant of 0.007 g mg^−1^ min^−1^. An adsorption-desorption equilibrium was reached at 120 min, suggesting that there is low affinity of PP towards the acidic dye. The rate-limiting step of the adsorption process was intraparticle diffusion of external mass transfer. The vibrational spectroscopic analysis suggested that AB25 is bound to the PP surface via electrostatic and hydrogen bonding interactions. The adsorption mechanism could be explained by the Langmuir isotherm model, demonstrating that the AB25 adsorption took place and distributed homogeneously as a monolayer on the PP surface, with a maximum adsorption capacity of 26.9 mg g^−1^. This is comparable to many values reported for other adsorbents derived from agricultural wastes. The low adsorption capacity of AB25 can be explained by proposing that there is a low affinity of the negatively charged PP surface towards the anionic dye. The results could also be an indication that the adsorption of dyes strongly depends on their interactions, via electrostatic and hydrogen bonding, to functional groups rather than the BET surface area of the adsorption surface. The derived thermodynamic parameters suggested that AB25 was spontaneously adsorbed on the PP surface, and that the adsorption was endothermic and energetically more favorable at higher temperatures. The entropy change of AB25 adsorption on PP is much smaller than previously reported values on other agricultural waste adsorbates, suggesting that the PP surface is energetically more stable, compared with that of other agricultural wastes. These results all show that PP has great potential for use in extracting similar toxins from wastewaters, and that scale up to industrial levels would be appropriate to test this. This is motivated by the low cost of PP and its efficiency of action.

## Figures and Tables

**Table 1:** The adsorption parameters of AB25 adsorption on PP deduced from the best fits of the empirical Langmuir, Freundlich, Dubinin–Radushkevich, and Temkin isotherm models to the experimental data.

Isotherm Model	Parameters	Quantity
Langmuir		
	$Q_{m}$ (mg g^−1^)	26.9
	$K_{L}$ (dm^−3^ mmol^−1^)	3.36
	R^2^	0.993
Freundlich		
	$K_{F}$	39.7
	n	1.26
	R^2^	0.998
Dubinin–Radushkevich		
	$Q_{m}$ (mg g^−1^)	20.3
	β	28.6
	E (kJ mol^−1^)	0.132
	R^2^	0.914
Temkin		
	$K_{T}$ (dm^−3^ mmol^−1^)	2.95
	$b_{T}$ (kJ mol^−1^)	0.354
	R^2^	0.758

**Table 2:** Adsorption on PP (current study) and data reported for other adsorbents in the literature.

Adsorbent	pH_pzc_	pH	Q_m_ (mg g^−1^)	Reference
Raw kaolin	5.70	7.3	16.5	[37]
*Shorea dasyphylla* sawdust	8.25	2.0	24.4	[24]
Pine sawdust	2.3–3.8	2.5–4.2	26.2	[15]
Cempedak durian peel	4.01	2	26.6	[25]
Pomelo pith	4.23	6.7	26.9	This study
Oak sawdust	2.3–3.8	2.5–4.2	27.9	[15]
Rubber leaves powder	NR *	2	28.1	[29]
*Nephelium lappaceum* Linn. seed	6.2	2	35.6	[26]
Walnut sawdust	2.3–3.8	2.5–4.2	37.0	[15]
Soybean waste	NR *	2	38.3	[31]
*Azolla pinnata*	NR *	2	50.5	[31]
Hazelnut shell	2.3–3.8	2.5–4.2	60.2	[15]
Banana peel	6.32	6.7	70.0	[11]
*Ficus rasemosa* leaves powder	NR *	2	83.3	[30]
Durian peel	6.18	6.7	89.7	[11]
Peach seed powder	NR *	2	95.2	[28]
*Aspergillus oryzae*	NR *	2	105.3	[38]
Egg shell modified activated carbon	5.7	5.2–5.7	109.8	[23]
*Ceratophylum demersum*	NR *	2	129.7	[14]
*Potamogeton pusillus*	NR *	2	183.5	[14]
Waste tea activated carbon	7.2	7–11	203.3	[22]
Pectin derived from pomelo peel	2.63	6.7	739.0	[21]
Chitosan-activated carbon composite	NR *	4	909.1	[39]

* NR = not reported.

**Table 3:** The thermodynamic parameters of AB25 adsorption on PP.

Adsorbent	T (K)	K_L_	$K_{e}^{0}$	ΔG_ads_	ΔH	ΔS
		(kJ mol^−1^)		(kJ mol^−1^)	(kJ mol^−1^)	(J mol^−1^ K^−1^)
	293	3.23	7420	−4.77		
	298	3.36	7728	−4.95		
PP	303	3.95	9078	−5.43	10.4	52
	308	3.96	9106	−5.53		
	313	4.22	9683	−5.77		

## Data Availability

Not applicable.

## Supplementary Materials

The following supporting information can be downloaded online, Figure S1: The adsorption efficiency (η) of 20 cm^3^ AB25 (1.20 × 10^−4^ mol dm^−3^) in the presence of different dosages of PP from 25 mg to 300 mg, Figure S2: Plot of $\Delta G_{\mathrm{ads}}$ against temperature based on adsorption of 20 mL AB25 (1.20 × 10^−4^ mol dm^−3^) in the presence of 25 mg of PP.

## Sample Availability

Samples of the compounds are not available from the authors.
