# Supplementary material for: Adsorption Behavior and Dynamic Interactions of Anionic Acid Blue 25 on Agricultural Waste
Source: Molecules. 2022 Mar 6;27(5):1718. doi: 10.3390/molecules27051718 (PMC8911607; doi:10.3390/molecules27051718)
Supplement: Supplementary file 1 [file molecules-27-01718-s001.zip › molecules-1606117-supplementary.pdf]

Supplementary Information

Adsorption Behavior and Dynamic Interactions of Anionic Acid Blue 25 on Agricultural Waste

Ensan Waatriah ES Shahrin, Nur Alimatul Hakimah Narudin, Nurulizzatul Ningsheh M. Shahri, Sera Budi Verinda, Muhammad Nur, Jonathan Hobley, Anwar Usman

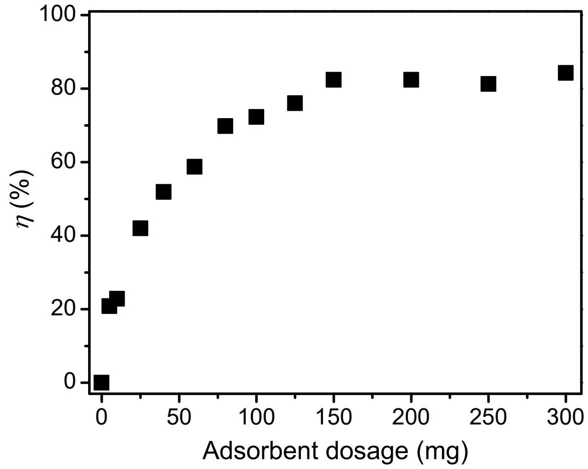

Figure S1. The adsorption efficiency (η) of AB25 on PP at different adsorbent dosages

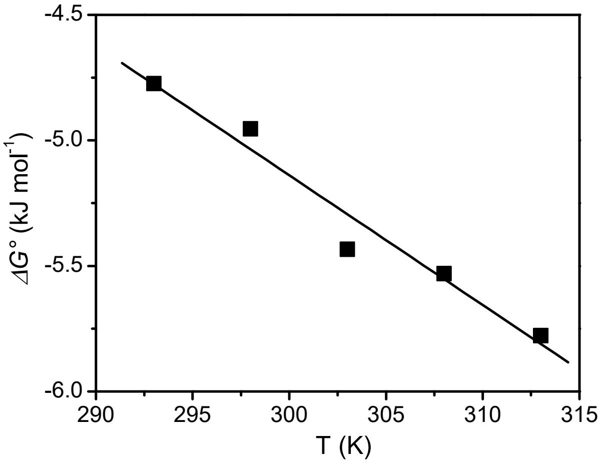

Figure S2. Plot of ΔG_{ads} against temperature, T.
